# Double Closed-Loop Obstruction Caused by Right Paraduodenal Hernia With Intestinal Malrotation

**DOI:** 10.7759/cureus.112887

**Published:** 2026-07-17

**Authors:** Yasuyuki Okabe, Kaoru Sakamoto, Tetsuya Shimada, Shinnosuke Hotta, Katsunari Sato

**Affiliations:** 1 Department of Surgery, Tsuruoka Municipal Shonai Hospital, Tsuruoka, JPN

**Keywords:** adult intestinal malrotation, closed-loop obstruction, double closed loop, right paraduodenal hernia, strangulated intestinal obstruction

## Abstract

Double closed-loop (DCL) obstruction is a rare but diagnostically important computed tomography (CT) finding observed in a subset of strangulated intestinal obstructions. A man in his 40s presented with sudden abdominal pain. CT demonstrated two simultaneous closed loop obstructions with a sac-like appearance, along with the superior mesenteric vein (SMV) rotation sign and absence of the duodenal horizontal segment, leading to a preoperative diagnosis of right paraduodenal hernia (RPDH) with intestinal malrotation. Emergency laparotomy was performed within three hours. Two closed loops of small bowel formed simultaneously, one inside and one outside a 3 cm hernial orifice on the dorsal ileocecal mesentery. Both loops resolved after reduction; the orifice was repaired without bowel resection. The patient was discharged on postoperative day 8. DCL obstruction is a rare but diagnostically valuable CT finding requiring prompt surgical intervention.

## Introduction

Double closed-loop (DCL) obstruction is an extremely rare CT finding observed in a subset of strangulated intestinal obstructions, in which two independent closed loops (CLs) form simultaneously around a single pivotal point [1]. A CL obstruction is a form of mechanical small bowel obstruction in which a segment of bowel is occluded at two points, creating an isolated, non-decompressible loop. Because DCL places a substantial length of bowel at risk of ischemia, it is a critical imaging finding that may necessitate extensive intestinal resection. Consequently, its recognition at the time of initial imaging carries high diagnostic value.

Right paraduodenal hernia (RPDH) is a type of internal hernia that arises from incomplete intestinal malrotation. Although paraduodenal hernias account for approximately 53% of all internal hernias, internal hernias themselves are responsible for fewer than 1% of all intestinal obstructions [2,3], making RPDH an uncommon cause of strangulated obstruction. RPDH has not previously been reported as a cause of DCL obstruction.

Here, we report what is, to our knowledge, the first documented case of DCL obstruction caused by RPDH with intestinal malrotation, describe the mechanism by which DCL arises in this setting, and discuss other conditions known to produce DCL.

This case report does not require ethical committee approval. Patient information has been de-identified, and written informed consent for publication was obtained from the patient.

## Case presentation

Patient history

A man in his 40s with no significant medical history presented to the emergency department with sudden-onset diffuse abdominal pain of two-hour duration. He reported similar, self-limiting episodes of abdominal pain occurring approximately once a year for the past several years, which may represent prior episodes of intermittent internal herniation with spontaneous reduction.

Physical examination

On arrival, the patient was alert and oriented. Vital signs were as follows: blood pressure 107/66 mmHg, heart rate 48 bpm, respiratory rate 24/minute, SpO2 98% on room air, and body temperature 36.8°C. The resting heart rate of 48 bpm was consistent with the patient's known baseline sinus bradycardia and was not considered clinically significant. Abdominal examination revealed diffuse tenderness without distension. No guarding, rebound tenderness, or rigidity was present, indicating the absence of overt peritoneal signs at the time of presentation.

Laboratory findings

Laboratory testing on arrival showed no evidence of inflammation, coagulopathy, renal dysfunction, or metabolic acidosis (Table 1). Notably, normal inflammatory markers and a normal lactate level do not exclude strangulated obstruction or early bowel ischemia.

**Table 1:** Laboratory Findings on Admission ALT, alanine aminotransferase; APTT, activated partial thromboplastin time; AST, aspartate aminotransferase; PaCO2, partial pressure of arterial carbon dioxide; PaO2, partial pressure of arterial oxygen; PT-INR, prothrombin time-international normalized ratio

Parameter	Value	Reference range
White blood cell count	7.2 × 10³/µL	3.3-8.6 × 10³/µL
Red blood cell count	4.84 × 10⁶/µL	4.35-5.55 × 10⁶/µL
Hemoglobin	15.7 g/dL	13.5-17.5 g/dL
Platelet count	209 × 10³/µL	158-348 × 10³/µL
Sodium	139 mEq/L	136-145 mEq/L
Potassium	3.9 mEq/L	3.6-4.8 mEq/L
Blood urea nitrogen	16.2 mg/dL	8-20 mg/dL
Creatinine	1.10 mg/dL	0.65-1.07 mg/dL
Total bilirubin	1.2 mg/dL	0.4-1.5 mg/dL
AST	28 U/L	13-40 U/L
ALT	15 U/L	10-42 U/L
C-reactive protein	0.03 mg/dL	<0.20 mg/dL
APTT	27.1 seconds	25-39 seconds
PT-INR	1.10	0.85-1.15
Arterial pH (room air)	7.359	7.35-7.45
PaCO2	34 mmHg	35-45 mmHg
PaO2	87 mmHg	83-108 mmHg
Lactate	0.7 mmol/L	0.5-2.2 mmol/L

Imaging findings

**Figure 1:**
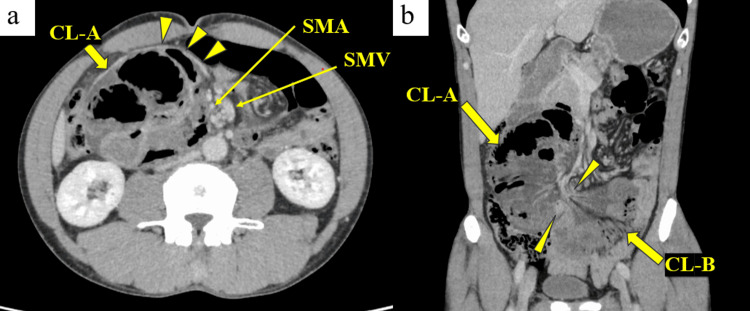
Contrast-Enhanced Computed Tomography (a) CT revealed dilated small bowel loops with diminished mural enhancement in the right upper abdomen, forming a CL obstruction (CL-A) with a sac-like appearance suggestive of encasement within a peritoneal sac (arrow). These loops displaced the SMV anteriorly from the dorsal aspect (arrowhead). The SMV was located to the left of the SMA (SMV rotation sign). (b) A second CL obstruction (CL-B), consisting of dilated loops with reduced mural enhancement, was identified in the left lower quadrant (arrow). The two narrowing points of CL-A and CL-B converged at a single location (arrowhead). CL, closed loop; SMA, superior mesenteric artery; SMV, superior mesenteric vein

Contrast-enhanced CT of the chest, abdomen, and pelvis was performed one hour after arrival (two hours after symptom onset), using intravenous contrast in the portal venous phase with multiplanar reconstructions including coronal and sagittal planes (Figure 1).

In the right upper abdomen, a cluster of dilated small bowel loops with diminished wall enhancement was identified, forming a CL obstruction (CL-A) with a sac-like appearance, consistent with encasement within a peritoneal sac (Figure 1a). These loops displaced the SMV anteriorly. A second dilated loop with reduced mural enhancement was identified in the left lower quadrant (CL-B) (Figure 1b). The two narrowing points of CL-A and CL-B converged at a single location. Mesenteric edema was identified adjacent to both CL-A and CL-B. The SMV was located to the left of the superior mesenteric artery (SMV rotation sign). The duodenum followed an aberrant course along the most posterior aspect of the sac and showed a beak sign at the single convergence point. A small amount of ascites was present. No pneumatosis intestinalis or portal venous gas was identified. The colon was in a normal anatomical position.

Based on the absence of a horizontal duodenal segment, the positional relationship of the SMV, the sac-like appearance, and the normal colonic position, a preoperative diagnosis of RPDH secondary to incomplete intestinal malrotation was established, and emergency laparotomy was planned.

Operative findings

**Figure 2:**
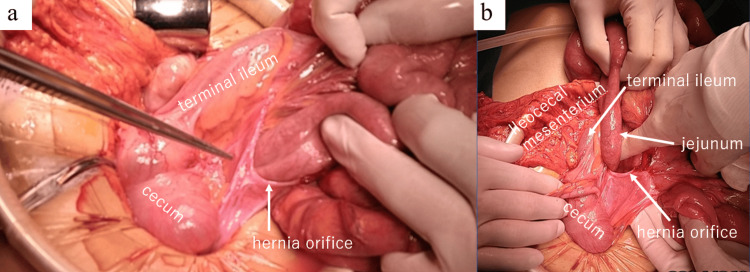
Intraoperative Findings (a) The proximal jejunum exited the retromesocolic space through a 3-cm hernial orifice located on the dorsal aspect of the ileocecal mesentery. (b) The segment of jejunum 60-140 cm distal to the jejunal origin was incarcerated through this orifice into an abnormal space posterior to the right mesocolon.

Surgery commenced five hours after symptom onset (three hours after admission). Laparotomy revealed a small amount of chylous ascites and dilated small bowel loops in the lower abdomen. The chylous nature of the ascites was identified by its characteristic milky appearance on direct visual inspection at laparotomy. The colon was in a normal position; however, the ligament of Treitz was absent, consistent with the preoperative CT finding of an absent horizontal duodenal segment and confirming the diagnosis of intestinal malrotation. The proximal jejunum exited the retromesocolic space through a 3-cm hernial orifice located on the dorsal aspect of the ileocecal mesentery (Figure 2a). The segment of jejunum 60-140 cm distal to the jejunal origin was incarcerated through this orifice into an abnormal space posterior to the right mesocolon (Figure 2b).

The proximal jejunum (0-60 cm) was compressed and narrowed by the incarcerated bowel, forming CL-B. The incarcerated bowel showed only mild venous congestion and lymphedema. Both CLs resolved immediately upon reduction of the incarcerated bowel. Bowel viability was confirmed intraoperatively based on prompt return of normal color and the presence of active peristalsis. Intestinal resection was therefore deemed unnecessary. Since the colon was in a normal position and no Ladd's bands were identified, only primary closure of the hernial orifice and appendectomy were performed.

Postoperative course

Oral intake was resumed on postoperative day 2, and the patient was discharged home on postoperative day 8. Over six months of postoperative outpatient follow-up based on clinical assessment only, neither hernia recurrence nor intestinal stricture was observed, and the patient remained free of recurrent abdominal pain.

## Discussion

Intestinal malrotation encompasses a spectrum of anatomical anomalies arising from disturbances in the rotation and fixation of the midgut during embryonic development, ranging from complete failure of rotation with absent retroperitoneal fixation to cases in which rotation proceeds normally yet retroperitoneal fixation fails to occur [4]. Paraduodenal hernias arise from congenital peritoneal recesses adjacent to the duodenum and account for the majority of internal hernias [2,3]. They have a left-to-right predominance of approximately 3:1 and a male-to-female ratio of 2:1 [5]. RPDH specifically results from incomplete intestinal malrotation, in which impaired mesocolon fusion creates an abnormal retromesocolic space [4]. Intestinal malrotation occurs in approximately one in 10,000 live births; symptomatic presentation in adults is exceedingly rare, occurring in fewer than 1% of cases [4].

Stringer and Babyn classified intestinal malrotation into three main types and eight subtypes based on the stage at which embryonic rotation is arrested [6,7]. In the present case, the rotation anomaly was limited to the duodenum while the colon maintained a normal position, a pattern consistent with type IIA in Stringer's classification, which is defined by isolated nonrotation of the duodenum with normal colonic rotation. Accurate preoperative diagnosis of RPDH relies on familiarity with its embryological basis and recognition of characteristic CT features. The reported preoperative diagnostic rate for paraduodenal hernias is as low as 43% [8], and contrast-enhanced CT is essential. Key CT findings include the following: (i) malposition of the colon; (ii) SMV rotation sign, indicating leftward displacement of the SMV relative to the superior mesenteric artery; (iii) sac-like appearance of the incarcerated bowel loops; and (iv) anterior displacement of the superior mesenteric vessels [9]. Notably, in the present case, the colon occupied a normal anatomical position, representing an atypical feature of incomplete intestinal malrotation in which mesocolon fusion was partially achieved. This atypical colonic position may reduce diagnostic confidence; however, the combination of the remaining CT findings - absent horizontal duodenal segment, SMV rotation sign, and sac-like bowel cluster - was sufficient to establish a correct preoperative diagnosis. Systematic evaluation of these features may improve the likelihood of reaching a correct preoperative diagnosis.

**Figure 3:**
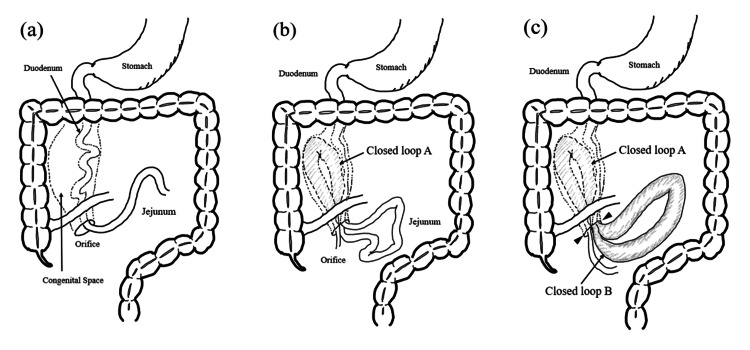
Schematic Diagram Illustrating the Mechanism of Double Closed-Loop Obstruction in Right Paraduodenal Hernia (a) Baseline anatomy: the duodenum descends without forming a horizontal segment and exits into the free peritoneal cavity through a hernial orifice on the dorsal aspect of the ileocecal mesentery. A congenital retromesocolic space is present posterior to the right mesocolon. (b) The jejunum (60-140 cm from the jejunal origin) herniates through the orifice into the retromesocolic space, forming CL-A and producing strangulated intestinal obstruction. (c) Constriction at the hernial orifice (arrowhead) compresses the proximal jejunum (0-60 cm), forming a second CL-B in the free peritoneal cavity. CLs are thus present both inside and outside the hernial sac, centered around the hernial orifice, completing the double CL configuration. This schematic is an original illustration hand-drawn by the first author. CL, closed loop

The mechanism by which RPDH generates DCL obstruction in the present case is illustrated in Figure 3.

Under normal anatomical conditions in this patient, the duodenum descended without forming a horizontal segment and re-entered the free peritoneal cavity through the hernial orifice on the dorsal aspect of the ileocecal mesentery (Figure 3a). When a segment of jejunum (60-140 cm distal to the jejunal origin) herniated into the retromesocolic space, it produced CL-A (Figure 3b). Critically, the hernial orifice had already been traversed once by the duodenum in the proximal direction. Enlargement of the incarcerated loop caused compression and narrowing of the hernial orifice, which in turn strangulated the proximal jejunum (0-60 cm) and formed a second CL, CL-B (Figure 3c). Therefore, CL-A (inside the hernial sac, exhibiting sac-like appearance) and CL-B (in the free peritoneal cavity, identified in the left lower quadrant) coexisted, producing DCL obstruction. The fundamental prerequisite for DCL formation in this context is that the proximal bowel passes through the hernial orifice before the incarceration; among all types of internal hernias, this anatomical configuration is unique to RPDH.

Despite its diagnostic importance, the term "double closed loop" appears in only a small number of published reports [1,10,11]. A literature search was conducted using PubMed and Ichushi-Web (Japan Medical Abstracts Society) with the search terms "double closed loop," "right paraduodenal hernia," and "intestinal malrotation," covering all articles published up to June 2025. No prior report of DCL obstruction caused by RPDH was identified.

Other conditions capable of producing DCL obstruction include intestinal knotting and Maydl's hernia. Intestinal knotting (also termed compound volvulus or intestinal knotting syndrome) is defined as a strangulating obstruction in which one loop of bowel wraps around another, forming two simultaneous CLs. The most common variant is ileosigmoid knotting; small bowel-to-small bowel knotting is rare. Preoperative diagnosis rates are low (16% for ileosigmoid knotting; 0% for small bowel variants) because these obstructions occur within the free peritoneal cavity, making anatomical localization difficult [12].

Maydl's hernia (double loop hernia) occurs when a W-shaped loop of bowel herniates through a relatively large abdominal wall or inguinal hernial orifice. Although rare, it can produce DCL obstruction, and the intra-abdominal component of the loop may be overlooked on CT if the finding is not specifically sought [13].

Although the identification of a CL on CT generally mandates urgent surgery for suspected strangulation regardless of the precise etiology, recognition of DCL provides additional critical information: the length of bowel at risk of ischemia is substantially greater, increasing the risk of extensive intestinal resection and worsening prognosis. Importantly, DCL does not necessarily indicate established bowel ischemia or necrosis, as illustrated by the present case in which bowel viability was preserved; rather, it signifies that a larger bowel segment is vulnerable and that the time window for intervention may be correspondingly narrower. In the present case, the correct preoperative diagnosis was established within one hour of arrival, surgery commenced within three hours, and hernial reduction was achieved five hours after symptom onset. While multiple factors influence bowel viability, including the degree and duration of strangulation, early operative intervention likely contributed to the avoidance of bowel resection in this case. When DCL obstruction is identified on CT, clinicians should promptly consider RPDH, intestinal knotting, and Maydl's hernia and pursue the most accurate preoperative diagnosis possible while minimizing time to operative intervention.

## Conclusions

We report what is, to our knowledge, the first documented case of DCL obstruction caused by RPDH with intestinal malrotation. DCL is a rare but diagnostically valuable CT finding. Awareness of its underlying causes - including RPDH, intestinal knotting, and Maydl's hernia - and prompt surgical intervention are essential to avoid extensive bowel resection.
